# Hierarchical packing of racemic metallosupra­molecular cages with Ni(II)-based triple-stranded helicate building blocks

**DOI:** 10.1107/S2052252523002385

**Published:** 2023-03-31

**Authors:** Thanh Nhan Nguyen, Anh Ngoc Nguyen, Ngoc Minh Tran, In-Hyeok Park, Hyojong Yoo

**Affiliations:** a Department of Materials Science and Chemical Engineering, Hanyang University, Ansan, Gyeonggi-do 15588, Republic of Korea; b Graduate School of Analytical Science and Technology (GRAST), Chungnam National University, Daejeon 34134, Republic of Korea; Sun Yat-Sen University, China

**Keywords:** nickel-based racemic cages, high-order assemblies, triple-stranded helicates, supramolecular building blocks, metallocavitands, metallosupramolecular cages

## Abstract

Three novel hierarchical Ni-based metallosupramolecular cages were constructed from nickel ions, pyridine di­carboxyl­ates and isophthalate derivative ligands (the substituents on C5 of isophthalate are methyl, *tert*-butyl and bromo groups). In every cage, two multinuclear nickel clusters, assembled from four nickel atoms and three pyridine di­carboxyl­ate ligands, are interlinked by three isophthalate-derivative ligands to form a nickel-based triple-stranded helicate (TSH), which then becomes the supramolecular building block for the fabrication of a metallocage. Six homochiral TSH supramolecular building blocks, either left (*M*)-handed or right (*P*)-handed, are connected by four linking nickel atoms to generate *M*_6_ and *P*_6_ discrete racemic cage molecules (*M*_6_ – cage with six *M*-TSHs; *P*_6_ – cage with six *P*-TSHs). The crystal packing of the racemic cages was characterized by single-crystal X-ray diffraction. An additional cobalt-based molecular cage with 5-methyl­isophthalate bridging ligands was synthesized for host–guest interaction studies. The methyl groups in Co- and Ni-TSH can act as guest units to be accommodated in the cone-shaped metal clusters (host) of an adjacent cage.

## Abstract 2

Racemic metallosupramolecular cages were fabricated from homochiral Ni(II)-based triple-stranded helicate building blocks. In hierarchical crystal packing, methyl groups of a cage can be accommodated in cone-shaped metal cluster metallocavitands of adjacent cages in the form of host–guest interactions.

## 1. Introduction

The fabrication of metal–organic platforms with high degrees of hierarchy, structural diversity and complexity has recently attracted significant interest in solid-state materials (Yoo *et al.*, 2015 ▸; Nouar *et al.*, 2008 ▸; Chakraborty *et al.*, 2021 ▸). To access these advanced architectures, employing coordination-driven self-assembly of predefined, well organized supramolecular building blocks (SBBs) as direct constructing units has proven to be a more effective method compared with the conventional approach of using molecular building blocks (MBBs), that is, multinuclear clusters and organic ligands (Chen *et al.*, 2015 ▸; Nouar *et al.*, 2008 ▸). Indeed, to create a high-order structure, suitable building units should possess a high degree of symmetry and connectivity which can be more feasibly obtained from SBBs than from MBBs (Nouar *et al.*, 2008 ▸). To date, several hierarchical architectures have been created by the coordination-driven self-assembly of SBBs (Perry IV *et al.*, 2009 ▸; Chen *et al.*, 2015 ▸; Nouar *et al.*, 2008 ▸). These SBBs are mostly assembled into one-dimensional polymers (Yoo *et al.*, 2015 ▸; Mai *et al.*, 2018 ▸; Hong *et al.*, 2000 ▸; Li *et al.*, 2011 ▸; Liu *et al.*, 2012 ▸; Sikligar *et al.*, 2021 ▸) or metal–organic frameworks (Pang *et al.*, 2014 ▸; Qian *et al.*, 2014 ▸; Li *et al.*, 2009 ▸; Tian *et al.*, 2014 ▸; Park *et al.*, 2007 ▸; Nouar *et al.*, 2008 ▸).

In supramolecular chemistry, the chirality investigation of discrete metallosupramolecules has received considerable attention due to its potential in chiral recognition, asymmetric catalysis, enantiomer separation and so on (Chen *et al.*, 2017 ▸). Chirality, in general, can be introduced into coordination platforms via two basic approaches: (1) a ‘hard’ approach harnesses optically pure chiral building blocks to generate predetermined chirality (Du *et al.*, 2022 ▸; Zhu *et al.*, 2022 ▸); (2) a ‘soft’ approach utilizes achiral building blocks to produce chirality (Wang *et al.*, 2022 ▸), and the chirality disappears on dissociation of metal–ligand bonds (Chen *et al.*, 2017 ▸). It was found that the second approach generally results in a racemic mixture (Zhang *et al.*, 2020 ▸; Wang *et al.*, 2020 ▸), both in solution and in the solid state since the chirality depends largely on metal coordination bonds, which are commonly labile in solution [thus allowing the interconversion of enantiomers (Chen *et al.*, 2017 ▸)].

Discrete metallosupramolecular cages (metallocages) with well defined cavities have been extensively studied over the past decades owing to their structural aesthetics (Yoshizawa *et al.*, 2009 ▸; McConnell, 2022 ▸; Yong *et al.*, 2022 ▸; Kim *et al.*, 2022 ▸) and intriguing applications in host–guest chemistry (Browne *et al.*, 2013 ▸; Jia *et al.*, 2020 ▸; Rizzuto *et al.*, 2019 ▸; Huang *et al.*, 2022 ▸), modification of the chemical reactivity of guests (Fang *et al.*, 2019 ▸; Whitehead *et al.*, 2013 ▸) [activating guests for reactions (Hastings *et al.*, 2010 ▸) or stabilizing reactive guests (Mal *et al.*, 2009 ▸; Galan & Ballester, 2016 ▸)], or gas storage (Duriska *et al.*, 2009 ▸). Most of these cages are built directly from MBBs, with a low degree of hierarchy and complexity (Pullen *et al.*, 2021 ▸; Lee *et al.*, 2023 ▸). Few studies have reported the fabrication of higher-order supramolecular cages using the SBB approach (Mai *et al.*, 2017 ▸; Kang *et al.*, 2018 ▸). These cage structures share similar features in terms of using cobalt-based triple-stranded helicates (TSHs) as SBBs for the formation of hierarchical cobalt-based supramolecular cages. In a single building block, cobalt ions and 2,6-pyridine di­carboxyl­ates (PDAs) were assembled into a tetrahedral cobalt cluster, and two clusters were interconnected by three isophthalate derivative bridging ligands to generate a TSH (Mai *et al.*, 2019 ▸; Tran & Yoo, 2020 ▸). Six TSHs were connected via four linking cobalt atoms to create a discrete cobalt-based supramolecular cage with a vast number of metal moieties and functional groups from organic ligands (Mai *et al.*, 2019 ▸, 2017 ▸).

To create more hierarchical supramolecular platforms with greater structural diversity and functionality for various purposes, Ni(II) salts were employed as the metal source, and three types of bridging ligands were employed for TSH formation, namely 5-methyl isophthalate (CH_3_-PTA), 5-*tert*-butyl isophthalate (*t*-butyl-PTA) and 5-bromo isophthalate (Br-PTA). Although Co(II) and Ni(II) were used for constructing TSHs (Le *et al.*, 2019 ▸), the ability of nickel-TSH to form metallocages has not been reported. Moreover, although *t*-butyl-PTA and Br-PTA have been employed for the formation of cobalt hierarchical cages from Co-TSH, the ability of CH_3_-PTA to form cages with high-order assemblies has not been reported. Herein, the successful syntheses of three new nickel-based supramolecular cages from smaller Ni-TSH building blocks with CH_3_-PTA, *t*-butyl-PTA and Br-PTA bridging ligands are reported. The coordination-driven self-assembly of six homochiral TSHs of either left (*M*)-handed or right (*P*)-handed configuration resulted in the formation of *M*_6_ and *P*_6_ cages in the form of a racemate (*M*_6_ – cage with six *M*-TSHs; *P*_6_ – cage with six *P*-TSHs). The crystal packing of the resulting racemic cages was fully characterized. To the best of our knowledge, this is the first study to report the synthesis of nickel-based supramolecular cages using the SBB approach. In particular, the ability to form host–guest interactions between the methyl groups on one cage and the metal cluster of an adjacent cage was verified.

## 2. Experimental

### 2.1. Materials

Nickel(II) nitrate hexahydrate [Ni(NO_3_)_2_·6H_2_O, 97%, Sigma–Aldrich], nickel(II) acetate tetrahydrate [Ni(OAc)_2_·4H_2_O, 98%, Sigma–Aldrich], cobalt(II) nitrate hexahydrate [Co(NO_3_)_2_·6H_2_O, 98%, Sigma–Aldrich], 2,6-pyridinedi­carb­oxy­lic acid (H_2_PDA, C_7_H_5_NO4, 99%, Sigma–Aldrich), 5-*tert*-butyl isophthalic acid (*t*-butyl-H_2_PTA, C_12_H_14_O_4_, 99%, Sigma–Aldrich), 5-methyl isophthalic acid (CH_3_-H_2_PTA, C_9_H_8_O_4_, 97%, Sigma–Aldrich), 5-bromo­isophthalic acid (Br-H_2_PTA, C_8_H_5_O_4_Br, 97%, Sigma–Aldrich), methanol (Samchun, 99.5%), *N*,*N*-di­methyl­formamide (DMF, 99.99%, Burdick & Jackson) and acetone (99.96%, Burdick & Jackson) were used as received.

### 2.2. Syntheses

#### 2.2.1. Synthesis of {[Ni_8_(PDA)_4_(H_0.33_PDA)_2_(CH_3_-PTA)_3_(DMF)_6_]_6_-[Ni(H_2_O)_3_]_4_·*x*solvent} (**1**)

DMF solutions of Ni(NO_3_)_2_·6H_2_O (1.2 ml, 0.05 *M*), H_2_PDA (0.6 ml, 0.05 *M*) and CH_3_-H_2_PTA (0.3 ml, 0.05 *M*) were mixed in a 4 ml glass vial at room temperature (RT). The vial was sealed, heated to 100°C (heating rate 2.67°C min^−1^), maintained for 24 h and then cooled to 30°C (cooling rate 0.25°C min^−1^). Complex **1** was obtained as green rectangular crystals. The solid yield was 44.3% based on H_2_PDA. Analytical calculation for C_486_H_456_N_60_O_274_Ni_52_ as {[Ni_8_(PDA)_4_(H_0.33_PDA)_2_(CH_3_-PTA)_2_(DMF)_4_(H_2_O)_2_]_6_-[Ni(H_2_O)_3_]_4_·(H_2_O)_10_}: calculated: C: 40.01, H: 3.13, N: 5.76; obtained: C: 39.71, H: 3.52, N: 5.36.

#### 2.2.2. Synthesis of {[Ni_8_(PDA)_4_(H_0.33_PDA)_2_(*t*-butyl-PTA)_3_(DMF)_4_(H_2_O)_2_]_6_-[Ni(H_2_O)_3_]_4_·*x*solvent} (**2**)

DMF solutions of Ni(OAc)_2_·6H_2_O (0.9 ml, 0.05 *M*), H_2_PDA (0.6 ml, 0.05 *M*) and *t*-butyl-H_2_PTA (0.3 ml, 0.05 *M*) were mixed in a 4 ml glass vial at RT. The vial was sealed, heated to 100°C (heating rate 2.67°C min^−1^), maintained for 24 h and then cooled to 30°C (cooling rate 0.25°C min^−1^). Complex **2** was obtained as green rectangular crystals. The solid yield was 32.7% based on H_2_PDA. Analytical calculation for C_540_H_588_N_60_O_286_Ni_52_ as {[Ni_8_(PDA)_4_(H_0.33_PDA)_2_(*t*-butyl-PTA)_3_(DMF)_4_(H_2_O)_2_]_6_-[Ni(H_2_O)_3_]_4_·(H_2_O)_22_}: calculated: C: 41.68, H: 3.78, N: 5.40; obtained: C: 41.25, H: 4.16, N: 5.56.

#### 2.2.3. Synthesis of {[Ni_8_(PDA)_4_(H_0.33_PDA)_2_(Br-PTA)_3_(DMF)_6_]_6_-[Ni(H_2_O)_3_]_4_·*x*solvent} (**3**)

DMF solutions of Ni(NO_3_)_2_·6H_2_O (0.9 ml, 0.05 *M*), H_2_PDA (0.6 ml, 0.05 *M*) and Br-H_2_PTA (0.3 ml, 0.05 *M*) were mixed in a 4 ml glass vial at RT. The vial was sealed, heated to 100°C (heating rate 2.67°C min^–1^), maintained for 24 h and then cooled to 30°C (cooling rate 0.25 °C min^–1^). The solid yield of the reaction was 42.8% based on H_2_PDA. Analytical calculation for C_468_H_394_N_60_O_270_Ni_52_Br_18_ as {[Ni_8_(PDA)_4_(H_0.33_PDA)_2_(Br-PTA)_3_(DMF)_4_(H_2_O)_2_]_6_-[Ni(H_2_O)_3_]_4_·(H_2_O)_6_}: calculated: C: 35.84, H: 2.51, N: 5.36; obtained: C: 36.28, H: 2.91, N: 5.33.

#### 2.2.4. Synthesis of {[Co_8_(PDA)_4_(H_0.33_PDA)_2_(CH_3_-PTA)_3_(DMF)_6_]_6_-[Co(H_2_O)_3_]_4_·*x*solvent} (**4**)

DMF solutions of Co(NO_3_)_2_·6H_2_O (1.2 ml, 0.05 *M*), H_2_PDA (0.6 ml, 0.05 *M*), CH_3_-H_2_PTA (0.3 ml, 0.05 *M*) and methanol (0.1 ml) were mixed in a 4 ml glass vial at RT. The vial was sealed, heated to 100°C (heating rate 2.67°C min^–1^), maintained for 24 h and then cooled to 30°C (cooling rate 0.25°C min^–1^). Complex **4** was obtained as purple rectangular crystals. The solid yield, calculated based on H_2_PDA, was 42.5%. Analytical calculation for C_510_H_490_N_54_O_278_Co_52_ as {[Co_8_(PDA)_4_(H_0.33_PDA)_2_(CH_3_-PTA)_3_(DMF)_3_(H_2_O)_3_]_6_-[Co(H_2_O)_3_]_4_·(CH_3_OH)_14_}: calculated: C: 41.13, H: 3.29, N: 5.08; obtained: C: 41.44, H: 3.37, N: 4.92.

### 2.3. Instrumentation

Thermogravimetric analysis (TGA) was conducted from RT to 600°C at a heating rate of 10°C min^−1^ under a nitro­gen atmosphere using a TA instrument STD Q600 analyser. Powder X-ray diffraction (PXRD) was conducted using synchrotron radiation (wavelength: 1.1 Å) in a focused beam configuration in the 2θ range 2–20° at 298 K. The simulated PXRD patterns were obtained from single-crystal X-ray diffraction (SCXRD) data using the *Mercury* software (version 3.8; Macrae *et al.*, 2020 ▸). X-ray photoelectron spectroscopy (XPS) measurements for **1** and **2** were performed on an R3000 spectrometer (VG SCIENTA, UK), and XPS measurements for **3** and **4** were performed on a K-ALPHA spectrometer (Thermo VG, UK) with monochromatic Al *K*α X-ray radiation as the X-ray source. Magnetic measurements of the compounds were conducted using a Quantum Design MPMS3 magnetometer in the temperature range 3 K ≤ *T* ≤ 300 K under an applied field of 1000 Oe.

### 2.4. X-ray crystallography

The light green crystals of **1** (0.172 × 0.132 × 0.128 mm), **2** (0.097 × 0.093 × 0.069 mm^3^) and **3** (0.246 × 0.241 × 0.197 mm), and the purple crystals of **4** (0.169 × 0.114 × 0.111 mm) were mounted on a MiTeGen MicroMount. Diffraction data for these crystals were collected at 100 K on a Rayonix MX225HS detector with an Si(111) double-crystal monochromator equipped with a synchrotron radiation source (0.70000 Å) at the 2D Supramolecular Crystallography Beamline (2D SMC), Pohang Accelerator Laboratory (PAL), Pohang, Republic of Korea. All calculations for the structure determination were performed using the *SHELXTL2018/3* package (Sheldrick, 2015 ▸).

## 3. Results and discussion

Treatment of 4 equivalents of Ni(NO_3_)_2_·6H_2_O, 2 equivalents of 2,6-pyridinedi­carb­oxy­lic acid (H_2_PDA) and 1 equivalent of 5-methyl isophthalic acid (CH_3_-H_2_PTA) in di­methyl­formamide (DMF) at 100°C for 24 h led to the formation of {[Ni_8_(PDA)_4_(H_0.33_PDA)_2_(CH_3_-PTA)_3_(DMF)_6_]_6_-[Ni(H_2_O)_3_]_4_·*x*solvent} (**1**) (Scheme 1).[Chem scheme1] 
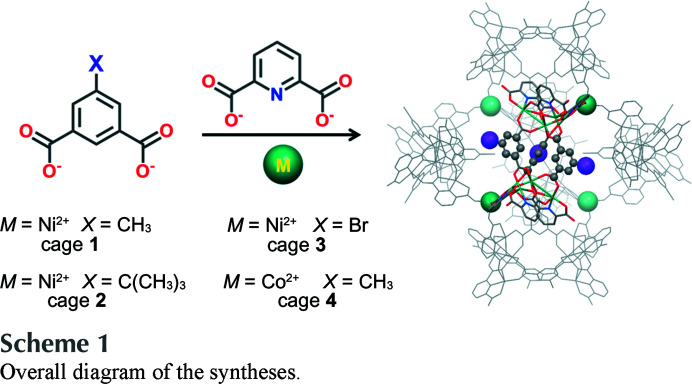


**Figure 1:**
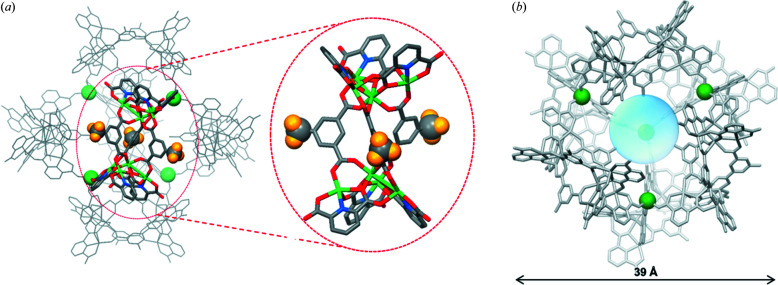
(*a*) X-ray crystal structure of **1** viewed along its *C*_2_ axis of symmetry. The four linking nickel atoms are illustrated by green balls. One TSH building block is highlighted and shown in the inset. Ni, C, O and N atoms are shown in green, grey, red and blue, respectively. All the disorder components, coordinated solvents and hydrogen atoms were omitted for clarity (with the exception of hydrogen atoms on the methyl groups of the highlighted TSH, which are presented by orange balls). (*b*) X-ray crystal structure of **1** viewed along its *C*_3_ axis of symmetry with its longest transverse distance and the confined space at the centre.

**Figure 2:**
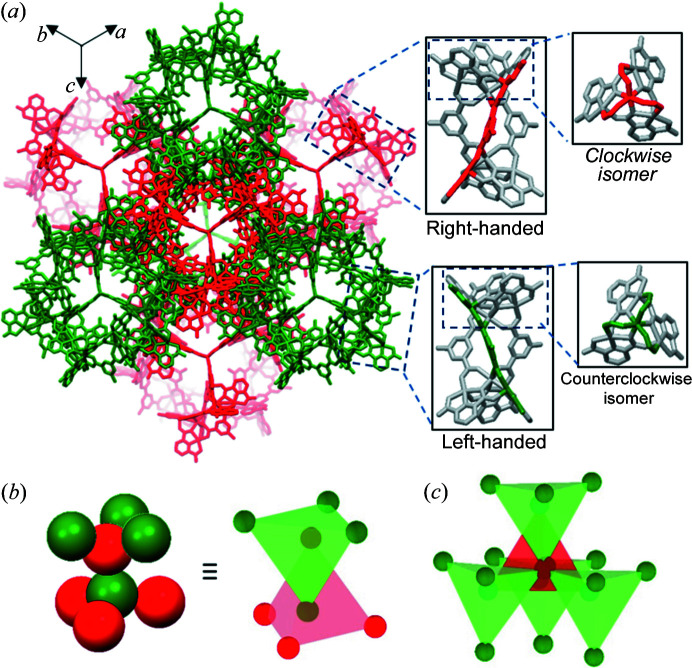
(*a*) Representation of a unit cell of **1** with *M*_6_ and *P*_6_ shown in green and red, respectively. (*b*) Illustration showing the arrangement of *M*_6_ (green balls) and *P*_6_ (red balls) in a unit cell with intertwisted *M*_6_- and *P*_6_-tetrahedron arrangements. (*c*) Illustration showing the arrangement of four *M*_6_ tetrahedrons at four vertexes of a *P*_6_ tetrahedron.

The solid-state structure of **1** was determined by SCXRD analysis and refined in the space group *Fd* 3 (Table 1 ▸). Discrete supramolecular cage **1**, comprised of six TSHs interconnected by four linking nickel atoms (Ni5), has the longest transverse distance of *ca* 39 Å (Figs. 1 ▸ and S1 of the supporting information). In a single Ni-TSH building block, each tetrahedral nickel cluster ({Ni_4_}), formed through the assembly of four nickel ions and three PDA ligands, is diagonally connected to another nickel cluster by three bridging CH_3_-PTA ligands. Each nickel cation in the cluster adopts a pseudooctahedral coordination environment. The nickel site at the centre is linked to three terminal nickel sites by three PDA ligands and linked to the other central nickel atoms by three CH_3_-PTA ligands. Each terminal nickel, in addition to coordinating with two PDA ligands and one CH_3_-PTA ligand, bonds with one DMF molecule [Figs. 1 ▸(*a*) and S1]. Note that the type of coordinated solvent (DMF or H_2_O) in terminal nickel atoms may not be the same in every experiment. Also, the PDA ligands are not completely deprotonated so as to balance the charge of the overall structure (as indicated in the chemical formula of **1**). Since the tetrahedral nickel clusters are assembled into either clockwise or counterclockwise isomers, the resulting TSHs, built from two metal clusters of the same chirality, can have either a left (*M*)-handed or right (*P*)-handed configuration [Fig. 2 ▸(*a*)]. Generally, the assembly of different chiral metal centres or clusters can generate helicates or mesocates (Albrecht, 2000 ▸; Nguyen *et al.*, 2022 ▸). Whether a helicate or mesocate is obtained depends heavily on the ligand rigidity (Albrecht, 2000 ▸). So far, all our TSHs obtained in which the bridging derivative ligands are short and rigid (a one-benzene ring system) are in helicate form (with either *M*- or *P*-configuration) (Yoo *et al.*, 2015 ▸; Mai *et al.*, 2017 ▸, 2018 ▸; Kang *et al.*, 2018 ▸; Le *et al.*, 2019 ▸).

For generating a discrete cage, six Ni-TSHs of the same chirality (homochiral) are required, and they are interconnected through the coordination with Ni5 atoms at the unoccupied carboxyl­ate oxygen atoms from the PDA ligands on the nickel cluster. Each Ni5, possessing a pseudo-octahedral coordination geometry, binds with three coordinated water molecules and three other TSHs through the unoccupied carboxyl­ate oxygen atoms in facial (*fac*) mode [Fig. S1(*a*)]. Four Ni5 atoms within a cage are arranged into a tetrahedron and separated from each other by a distance of 11.65 Å. The similar coordination-driven cage assemblies were previously reported in the solid-state structures in which the metal ion was cobalt and the substituents (on PTA ligands) were *t*-butyl, bromo and iodo groups (Mai *et al.*, 2017 ▸; Kang *et al.*, 2018 ▸). Since there are two configurations of TSH (*M-* and *P-*), two types of cage, *M*_6_ and *P*_6_ (*M*_6_ – cage with six *M*-TSHs; *P*_6_ – cage with six *P*-TSHs), can be obtained with opposite chiralities. Each metallocage possesses a confined space of a pseudo-regular tetrahedron with four Ni5 at each vertex, and an edge distance (Ni5⋯Ni5 separation) of 17.11 Å (Fig. S2). The void volume of **1**, including the inner void and the space between cages, calculated by *Olex2* is 119519.9 Å^3^ (Dolomanov *et al.*, 2009 ▸).

**Figure 3:**
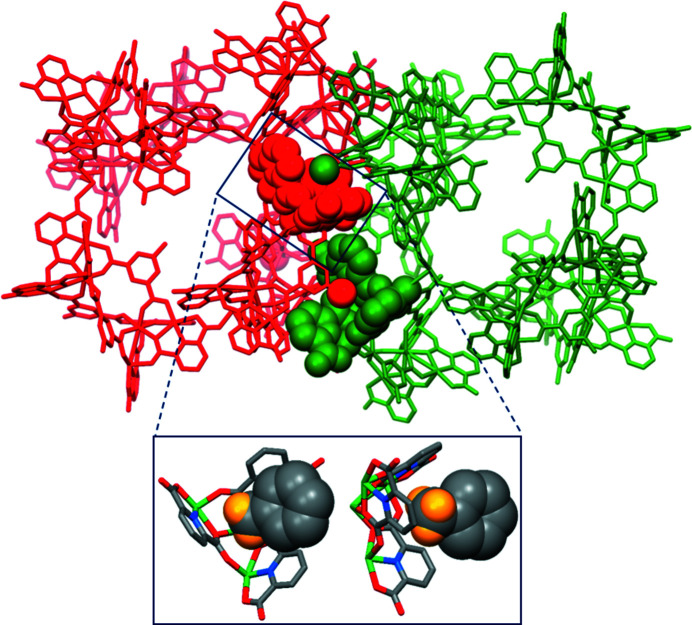
Two racemic cages of **1** with the host–guest interactions between the cone-shaped cavity of one molecular cage and the methyl group of the adjacent cage (shown in the inset).

The crystal packing of **1** indicated that each unit cell of **1** is built from eight cages packed together, of which four are *M*_6_ and four are *P*_6_ [Fig. 2 ▸(*a*)]. An *M*_6_ (or *P*_6_) is exposed to four other *P*_6_ (or *M*_6_) cages arranged at four vertices of a tetrahedron (Fig. S3). Thus, each unit cell of **1** is comprised of two intertwisted *M*_6_ and *P*_6_ tetrahedrons [Fig. 2 ▸(*b*)]. When considering a larger range, four *M*_6_ (or *P*_6_) cages at the vertices of an *M*_6_ (or *P*_6_) tetrahedron become the centres of the other *P*_6_ (or *M*_6_) tetrahedrons [Fig. 2 ▸(*c*)]. This assembly pattern is extended in three dimensions to create a well arranged crystal structure. In particular, the methyl group in TSH was found to have the ability to create host–guest interactions between neighbouring racemic cage molecules, in which the methyl group from one cage can be accommodated in a cone-shaped metal cluster of the adjacent cage (Fig. 3 ▸).

The phase purity of as-synthesized **1** was determined using PXRD (Fig. S4). The observed and expected PXRD patterns of **1** are considerably similar. The TGA curve of **1** exhibits a weight loss below 350°C attributed to the removal of trapped and coordinated solvents, and a sharp weight loss at approximately 400°C attributed to the decomposition of the organic ligands (Fig. S5) (Yoo *et al.*, 2015 ▸). XPS measurements confirmed the presence of Ni, C, O and N atoms in **1** [Fig. S6(*a*)]. The oxidation state of the nickel ions was determined to be +2 by the presence of two strong peaks at 856.7 and 874.2 eV correlating to Ni 2*p*_3/2_ and Ni 2*p*_1/2_, respectively [Fig. S6(*b*)] (Le *et al.*, 2019 ▸; Kang & Yoo, 2020 ▸). The bond valence sum calculation for the nickel ions also indicates a +2 oxidation state (Table S1) (IUCr, 2021 ▸; Brown, 2002 ▸). The temperature-dependent magnetization was measured to confirm the oxidation state of the metal ions in **1** using a Quantum Design MPMS3 magnetometer in the temperature range 3 K ≤ *T* ≤ 300 K under an applied field of 1000 Oe [Fig. S7(*a*)]. By applying the Curie–Weiss law for the fitting of 1/χ versus *T*, the Weiss constant was determined to be θ = −11.36 K for **1** [Fig. S7(*b*)], suggesting a weak antiferromagnetic interaction between metal ions (Hatscher *et al.*, 2005 ▸). The χ_M_ *T* value at 300 K was 1.42 emu K mol^−1^ for **1** [Fig. S7(*c*)], corresponding to μ_eff_ = 3.37 B.M, which is in the acceptable range of experimentally observed octahedral Ni(II) ions (Earnshaw, 1968 ▸).

Two other nickel-based molecular cages were also successfully synthesized using 5-*tert*-butyl isophthalic acid (*t*-butyl-H_2_PTA) and 5-bromo isophthalic acid (Br-H_2_PTA) as bridging ligands, denoted **2** and **3**, respectively. Specifically, 3 equivalents of Ni(OAc)_2_·4H_2_O, 2 equivalents of H_2_PDA and 1 equivalent of *t*-butyl-H_2_PTA in DMF were heated at 100°C for 24 h to form {[Ni_8_(PDA)_4_(H_0.33_PDA)_2_(*t*-butyl-PTA)_3_(DMF)_4_(H_2_O)_2_]_6_-[Ni(H_2_O)_3_]_4_·*x*solvent} (**2**). Similarly, the treatment of 3 equivalents of Ni(NO_3_)_2_·6H_2_O, 2 equivalents of H_2_PDA and 1 equivalent of Br-H_2_PTA in DMF at 100°C for 24 h afforded {[Ni_8_(PDA)_4_(H_0.33_PDA)_2_(Br-PTA)_3_(DMF)_6_]_6_-[Ni(H_2_O)_3_]_4_·*x*solvent} (**3**) (Scheme 1).

**Figure 4:**
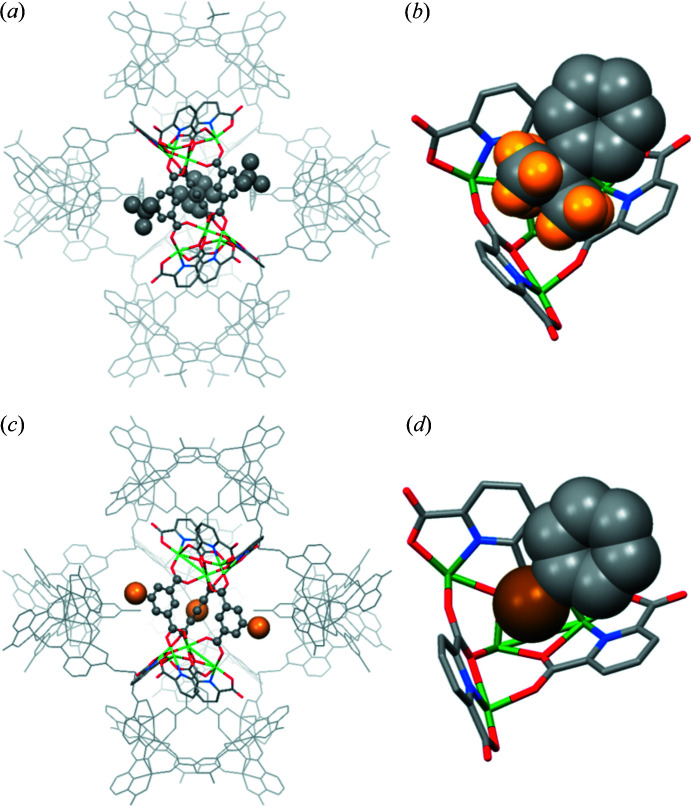
X-ray crystal structure of (*a*) **2** and (*c*) **3**. Host–guest interactions between two adjacent racemic cages of (*b*) **2** and (*d*) **3**. The balls in grey, yellow and brown represent C, H and Br, respectively.

The self-assembly processes of **2** and **3** are analogous to that of **1** with the interconnection of six homochiral TSH building blocks [Figs. 4 ▸(*a*) and 4 ▸(*c*)]. Their packing modes are alike with the intertwining of *M*_6_ and *P*_6_ tetrahedrons [Figs. S8(*a*), S8(*b*) and S8(*d*)]. The *tert*-butyl and bromo groups of the TSHs in **2** and **3** could also engage in the host–guest interactions between racemic cages [Fig. 4 ▸(*b*) and 4 ▸(*d*)]. The resemblance in the PXRD patterns of **1**, **2** and **3** (Fig. S4) further confirms that **1**–**3** are isomorphous. The TGA curves of **2** and **3** are not significantly different from that of **1**, and they indicate two main steps of chemical change: the removal of trapped and coordinated solvent molecules below 350°C and the ligand decomposition above 350°C (Fig. S5). XPS analysis [Figs. S6(*d*)–S6(*f*)], the calculated valence values (Table S1) and magnetic measurements [Figs. S7(*d*)–S7(*f*) and S7(*g*)–S7(*l*)] highlight that nickel ions in **2** and **3** have an oxidation state of +2. So far, we have been unable to obtain satisfactory UV–vis spectra of nickel-based supramolecular cages owing to the low solubility of these compounds in common organic solvents.

**Figure 5:**
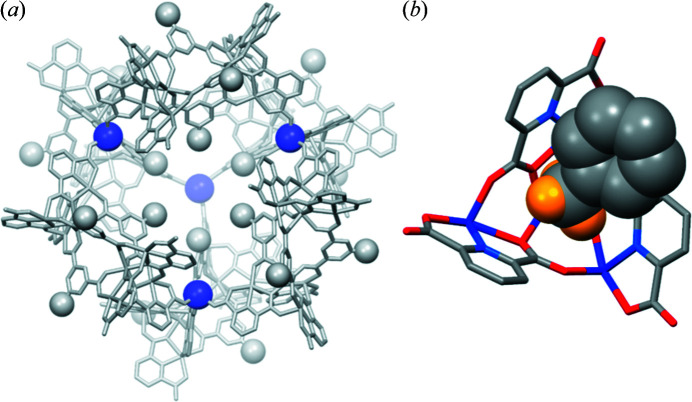
(*a*) X-ray crystal structure of **4**. The violet and grey balls represent Co and C atoms, respectively. (*b*) Host–guest interaction between a methyl group and cone-shaped cavity of two adjacent racemic cages.

To further investigate the ability of the methyl group to form host–guest interactions between racemic cages, a cobalt-based supramolecular cage was obtained on heating 4 equivalents of Co(NO_3_)_2_·6H_2_O, 2 equivalents of H_2_PDA and 1 equivalent of CH_3_-H_2_PTA in DMF with a small amount of methanol at 100°C for 24 h. The resulting cobalt cage {[Ni_8_(PDA)_4_(H_0.33_PDA)_2_(CH_3_-PTA)_3_(DMF)_6_]_6_-[Co(H_2_O)_3_]_4_·*x*solvent} was labelled **4**. The significant similarities in the single-crystal structure, the packing and the PXRD patterns of **4** and **1** indicate that **4** is isomorphous with **1** [Figs. 5 ▸(*a*), S4(*d*) and S8(*c*)]. The host–guest interaction between two racemic cages attributed to the methyl group observed in **1** is also present in **4** [Fig. 5 ▸(*b*)]. The oxidation state of cobalt (+2) in **4** was confirmed by the XPS spectra, the calculated valence values and the magnetic measurement results [Figs. S6(*g*)–S6(*h*) and S7(*j*)–S7(*l*), and Table S1].

Investigating the factors driving the formation of complexes **1**–**4** provides a deeper understanding of the cage assembly process, which is crucial for the rational design of advanced supramolecular cage architectures. The first factor favouring the generation of discrete molecular cages **1**–**4** is the availability of unoccupied carboxyl­ate oxygen atoms with suitable positions and orientations on the TSHs (Mai *et al.*, 2017 ▸). Considering one TSH building block, the two unoccupied oxygen atoms connected with the linking metal ion are located on the same ‘strand’ created by the extension of one bridging PTA ligand [Fig. S9(*a*)]. These two unoccupied oxygen atoms lie at almost opposite sides and set the longest distance that any two unoccupied oxygen atoms within a TSH can make [Fig. S9(*b*)]. With this coordination mode, the steric hindrance among neighbouring TSHs within a single cage can be minimized. The second contributing factor is related to the stability of the cages during the synthesis. Ni(II) and Co(II) ions, which differ in their own preferential coordination spheres and affinities to ligands, are expected to exhibit different coordination-driven assemblies (Le *et al.*, 2019 ▸; Housecroft & Sharpe, 2005 ▸). However, it was observed that both nickel and cobalt ions could adopt a *fac*-geometry mode to assemble six helicates and create a caged platform. It is therefore believed that the specific geometry of these supramolecular cages is energetically stable. In addition, since DMF was used as the solvent during the synthesis in the current case, its high polarity could cause the methyl/*tert*-butyl group in the TSHs to preferentially arrange in a manner that could minimize the exposure of nonpolar groups to polar solvent (Frischmann & Maclachlan, 2007 ▸; Rahman *et al.*, 2020 ▸). The last factor is the intermolecular host–guest interactions between racemic cages. Considering cage **3**, the electron-deficient tetranuclear metal cluster in one cage plays the role of a ‘metallocavitand’ to provide sufficient space for the accommodation of electronegative bromo groups of the adjacent cage [Fig. 4 ▸(*d*)]. The shortest Br⋯C9 distance in **3** was found to be 3.54 Å, which is slightly shorter than the sum of the van der Waals radii of C and Br (3.63 Å) (Rowland & Taylor, 1996 ▸). A similar interaction between an electron-deficient metal cluster acting as a host and electronegative bromo groups acting as guests has also been observed previously (Kang *et al.*, 2018 ▸). It was reported that six discrete TSHs, even without linking metal atoms, could be arranged circularly to generate a cage-like structure with a similar topology to those observed in cages **1**–**4**, and the driving force behind the cage-like assembly was the host–guest interaction (Kang *et al.*, 2018 ▸). These host–guest interactions seem to present between racemic cages in the packing of **1**, **2** and **4** [Figs. 3 ▸, 4 ▸(*b*) and 5 ▸(*b*)]. In fact, the host–guest interactions of the *tert*-butyl group and the methyl group have not been observed before. This is mainly because the methyl and *tert*-butyl groups are less electronegative than the bromo group. In the current work, the scope of cage structures with unique packing geometry is expanded to the Ni-based cage (**1**) with CH_3_-PTAs, Ni-based cage (**2**) with *t*-butyl-PTAs and Co-based cage (**4**) with CH_3_-PTAs. We expect that the current examples will provide researchers with a better understanding of complex molecular cages with SBBs and an effective strategy to facilitate the rational construction of these assemblies for specific applications.

## 4. Conclusions

A series of nickel-based metallosupramolecular cages {[Ni_8_(PDA)_4_(H_0.33_PDA)_2_(CH_3_-PTA)_3_(DMF)_6_]_6_-[Ni(H_2_O)_3_]_4_·*x*solvent} (**1**), {[Ni_8_(PDA)_4_(H_0.33_PDA)_2_(*t*-butyl-PTA)_3_(DMF)_4_(H_2_O)_2_]_6_- [Ni(H_2_O)_3_]_4_·*x*solvent} (**2**) and {[Ni_8_(PDA)_4_(H_0.33_PDA)_2_(Br-PTA)_3_(DMF)_6_]_6_-[Ni(H_2_O)_3_]_4_·*x*solvent} (**3**), and a cobalt-based molecular cage {[Ni_8_(PDA)_4_(H_0.33_PDA)_2_(CH_3_-PTA)_3_(DMF)_6_]_6_-[Co(H_2_O)_3_]_4_·*x*solvent} (**4**) were newly synthesized. These cages exhibit analogous assembly patterns with six homochiral SBBs linked by four metal atoms for the generation of discrete *M*_6_ and *P*_6_ cage molecules in the form of a racemate. The SCXRD data of **1**–**4** indicate that they possess similar packing behaviours, in which four neighbouring homochiral cages are arranged in four vertices of a tetrahedron with a cage of opposite chirality located at the centre of the tetrahedron. In addition, the packing structures of **1** and **4** indicate that the methyl group in the cobalt and nickel TSHs can be used for the host–guest interactions between racemic cages.

## Supplementary Material

Crystal structure: contains datablock(s) 1, 2, 3, 4. DOI: 10.1107/S2052252523002385/yc5042sup1.cif

Structure factors: contains datablock(s) 1. DOI: 10.1107/S2052252523002385/yc5042sup2.hkl

Structure factors: contains datablock(s) 2. DOI: 10.1107/S2052252523002385/yc5042sup3.hkl

Structure factors: contains datablock(s) 3. DOI: 10.1107/S2052252523002385/yc5042sup4.hkl

Structure factors: contains datablock(s) 4. DOI: 10.1107/S2052252523002385/yc5042sup5.hkl

Supporting tables and figures. DOI: 10.1107/S2052252523002385/yc5042sup6.pdf

CCDC references: 2209422, 2209425, 2209565, 2209566

## Figures and Tables

**Table 1:** Crystal data and structure refinements for **1**, **2**, **3** and **4**

	**1**	**2**	**3**	**4**
Formula	C_522_H_496_N_72_O_264_Ni_52_	C_540_H_544_N_60_O_264_Ni_52_	C_504_H_442_N_72_O_264_Ni_52_Br_18_	C_522_H_496_N_72_O_264_Co_52_
Formula weight	15054.80	15151.24	16222.57	15066.24
Crystal system	Cubic	Cubic	Cubic	Cubic
Space group	*Fd* 3	*Fd* 3	*Fd* 3	*Fd* 3
*a* (Å)	57.933 (7)	58.600 (7)	58.069 (7)	58.380 (7)
*b* (Å)	57.933 (7)	58.600 (7)	58.069 (7)	58.380 (7)
*c* (Å)	57.933 (7)	58.600 (7)	58.069 (7)	58.380 (7)
α (°)	90	90	90	90
β (°)	90	90	90	90
γ (°)	90	90	90	90
*V* (Å^3^)	194435 (67)	201234 (70)	195809 (68)	198973 (69)
*Z*	8	8	8	8
*D*_calc_(g cm^−3^)	1.029	1.000	1.101	1.006
μ (mm^−1^)	1.042	0.970	1.772	0.870
*F*(000)	61600.0	62176.0	65344.0	61184.0
Reflections collected	275607	319601	31600	311832
Independent reflections	15921 [*R*_int_ = 0.0722]	17258 [*R*_int_ = 0.0791]	16050 [*R*_int_ = 0.0137]	17071 [*R*_int_ = 0.0367]
GOF	1.053	1.025	1.026	1.067
*R*_1_, w*R*_2_ [*I* ≥ 2σ(*I*)]	0.0408, 0.1171	0.0500, 0.1555	0.0592, 0.1607	0.0413, 0.1144
*R*_1_, w*R*_2_ (all data)	0.0484, 0.1209	0.0639, 0.1650	0.0653, 0.1646	0.0457, 0.1171
CCDC nos.	2209422	2209565	2209425	2209566
