# Supplementary material for: Hierarchical packing of racemic metallosupra­molecular cages with Ni(II)-based triple-stranded helicate building blocks
Source: IUCrJ. 2023 Mar 31;10(Pt 3):321–8. doi: 10.1107/S2052252523002385 (PMC10161775; doi:10.1107/S2052252523002385)
Supplement: Supplementary file 6 [file m-10-00321-sup6.pdf]

IUCrJ

Volume 10 (2023)

Supporting information for article:

Hierarchical Packing of Racemic Metallosupramolecular Cages with Ni(II)-based Triple–Stranded Helicate Building Blocks

Thanh Nhan Nguyen, Anh Ngoc Nguyen, Ngoc Minh Tran, In-Hyeok Park and Hyojong Yoo

Table S1 The calculated valence values for nickel and cobalt ions in **1**, **2**, **3**, and **4** using the bond valence sum theory.

Compound	Atom	Bond valence sum
**1**	Ni1	2.14
	Ni2	2.09
	Ni3	2.10
	Ni4	2.11
	Ni5	2.05
**2**	Ni1	2.15
	Ni2	2.08
	Ni3	2.12
	Ni4	2.09
	Ni5	2.12
**3**	Ni1	2.14
	Ni2	2.12
	Ni3	2.13
	Ni4	2.10
	Ni5	1.94
**4**	Ni1	2.03
	Ni2	2.01
	Ni3	2.02
	Ni4	2.01
	Ni5	1.75

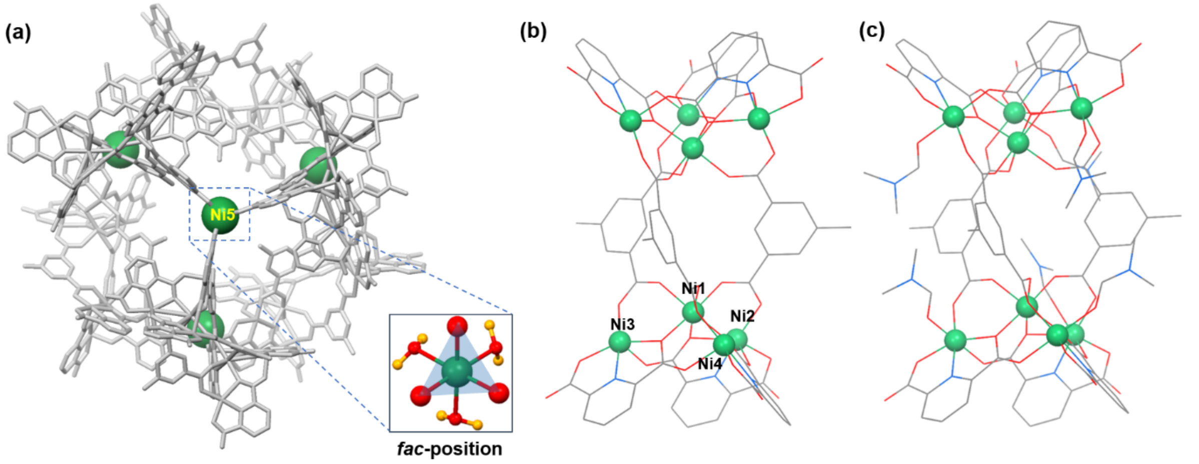

Figure S1 (a) Crystal structure of **1** with the inset illustrating the coordination mode of Ni5. (b) One TSH building block with the label of Ni atoms (coordinated solvents were omitted). (c) Full presentation of one TSH building block with coordinated solvents.

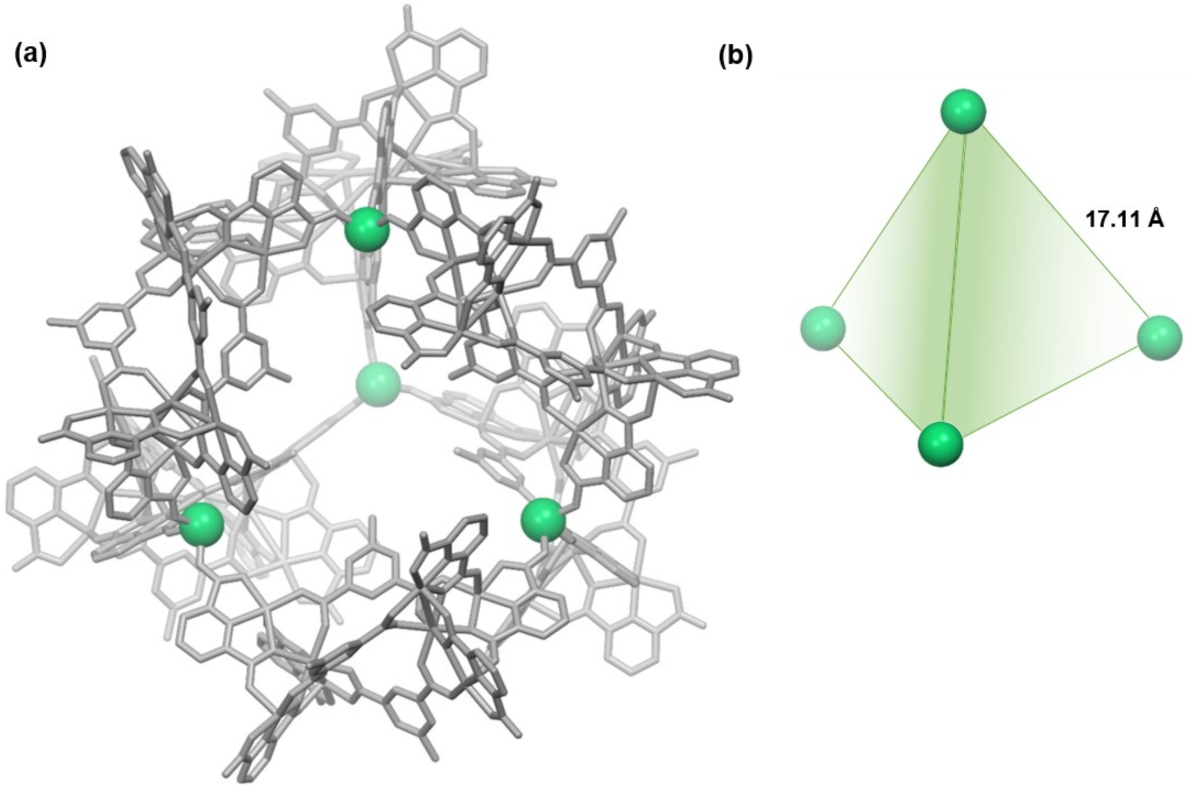

Figure S2 (a) Crystal structure of **1** with four green balls representing four Ni5 directed toward the cavity. (b) A large regular tetrahedron of **1** defined by four Ni5.

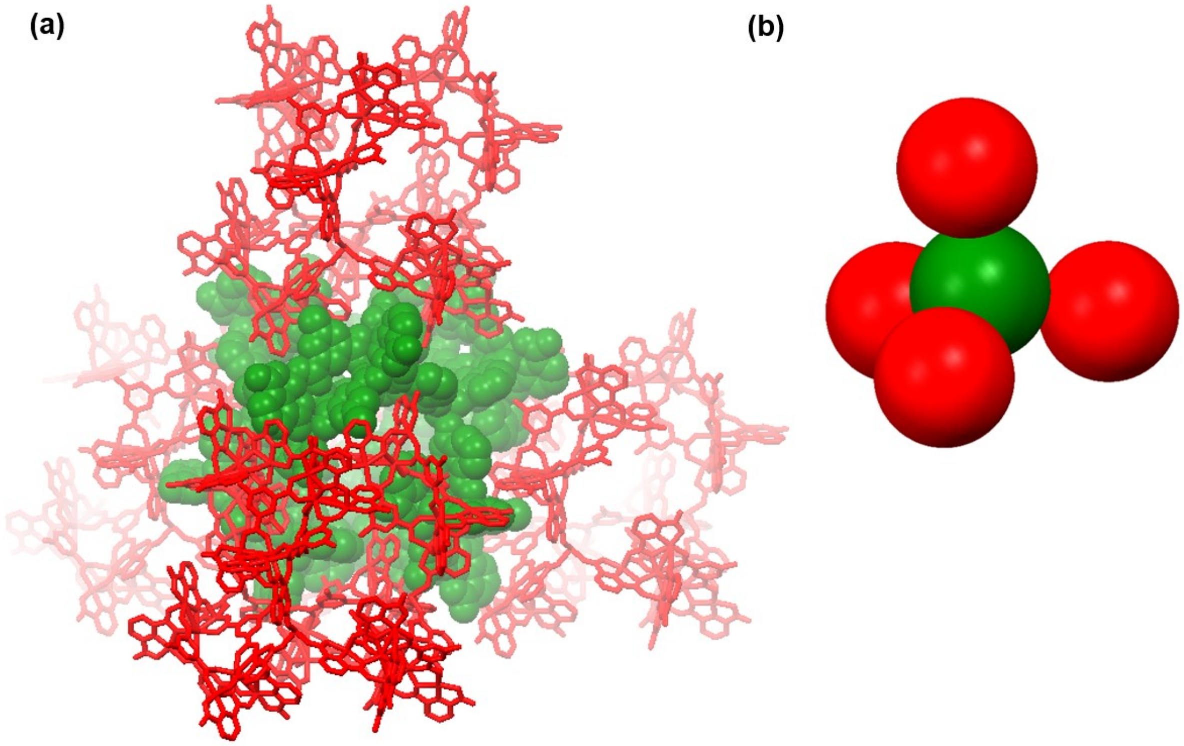

Figure S3 (a) One M_6 cage of **1** is exposed to four adjacent P_6 cages. (b) Cartoon representing the arrangement of four P_6 cages surrounding an M_6 cage.

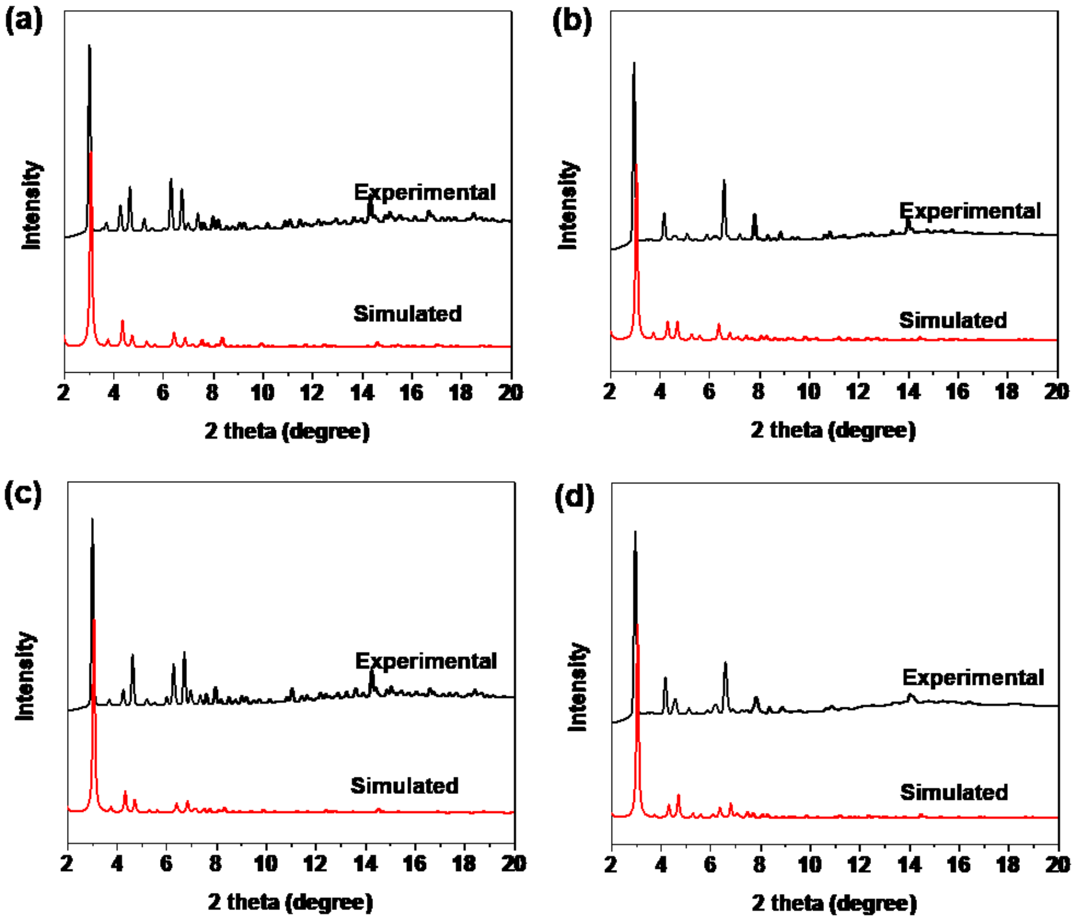

Figure S4 X-ray diffraction (PXRD) patterns of (a) complex **1**, (b) complex **2**, (c) complex **3**, and (d) complex **4**. Patterns simulated from the single-crystal structures are in red, and those obtained from the as-synthesized crystals are in black.

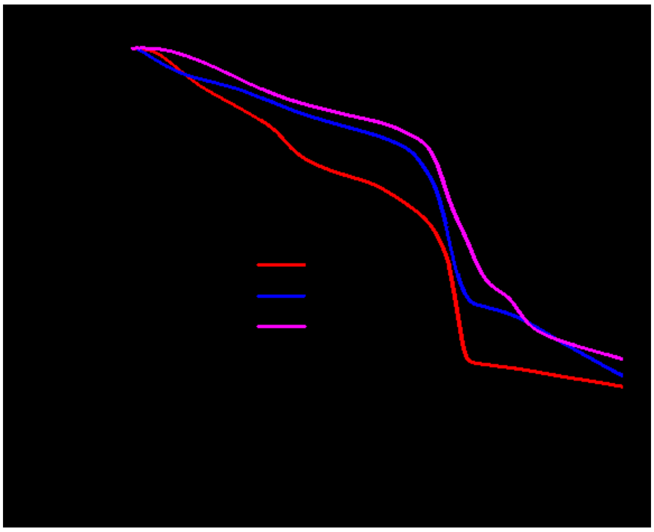

Figure S5 TGA curves of complexes **1**-**4**.

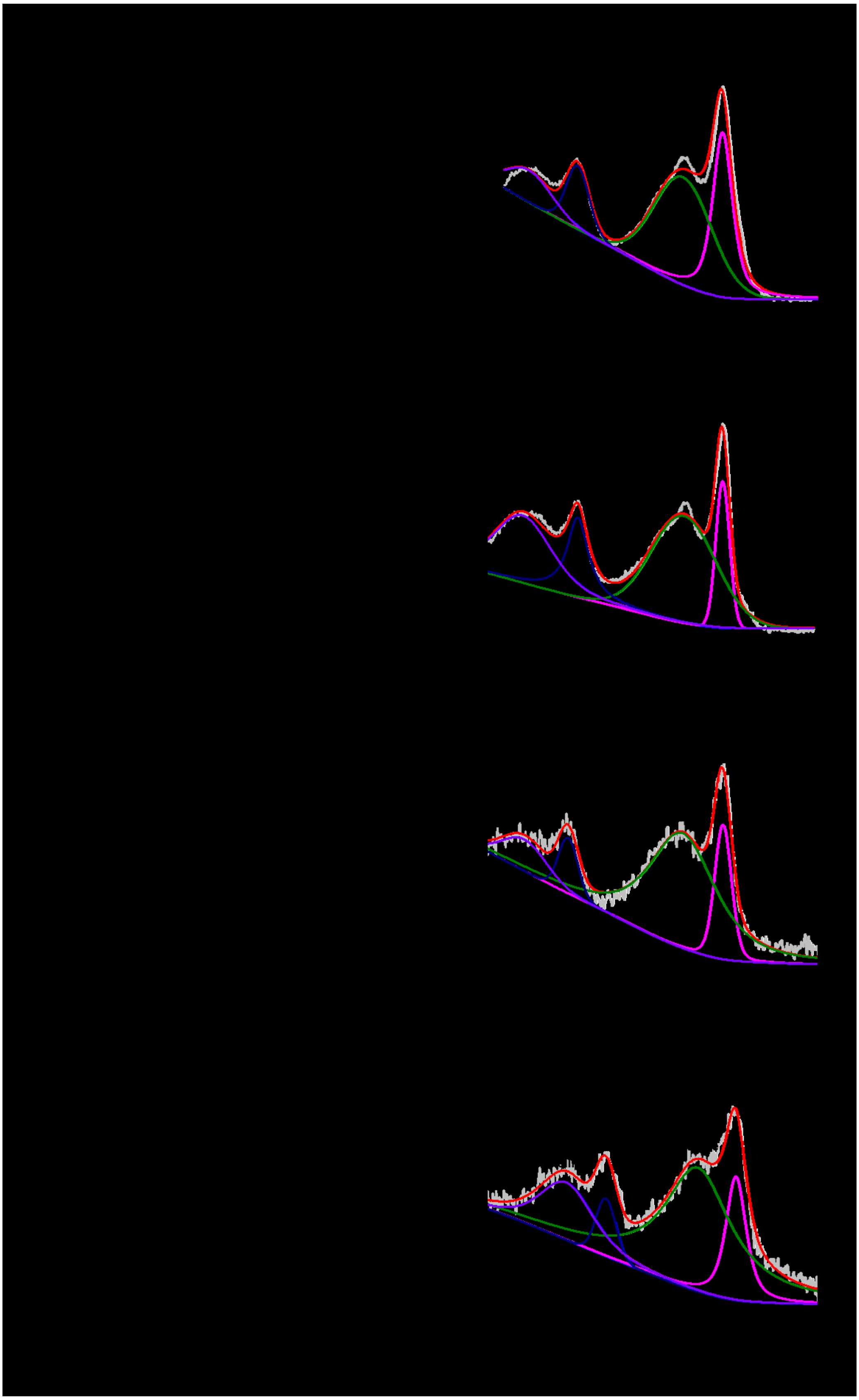

Figure S6 XPS full spectra and high resolution of Ni/Co2p XPS spectra of (a and b) complex **1**, (c and d) complex **2**, (e and f) complex **3**, and (g and h) complex **4**, respectively.

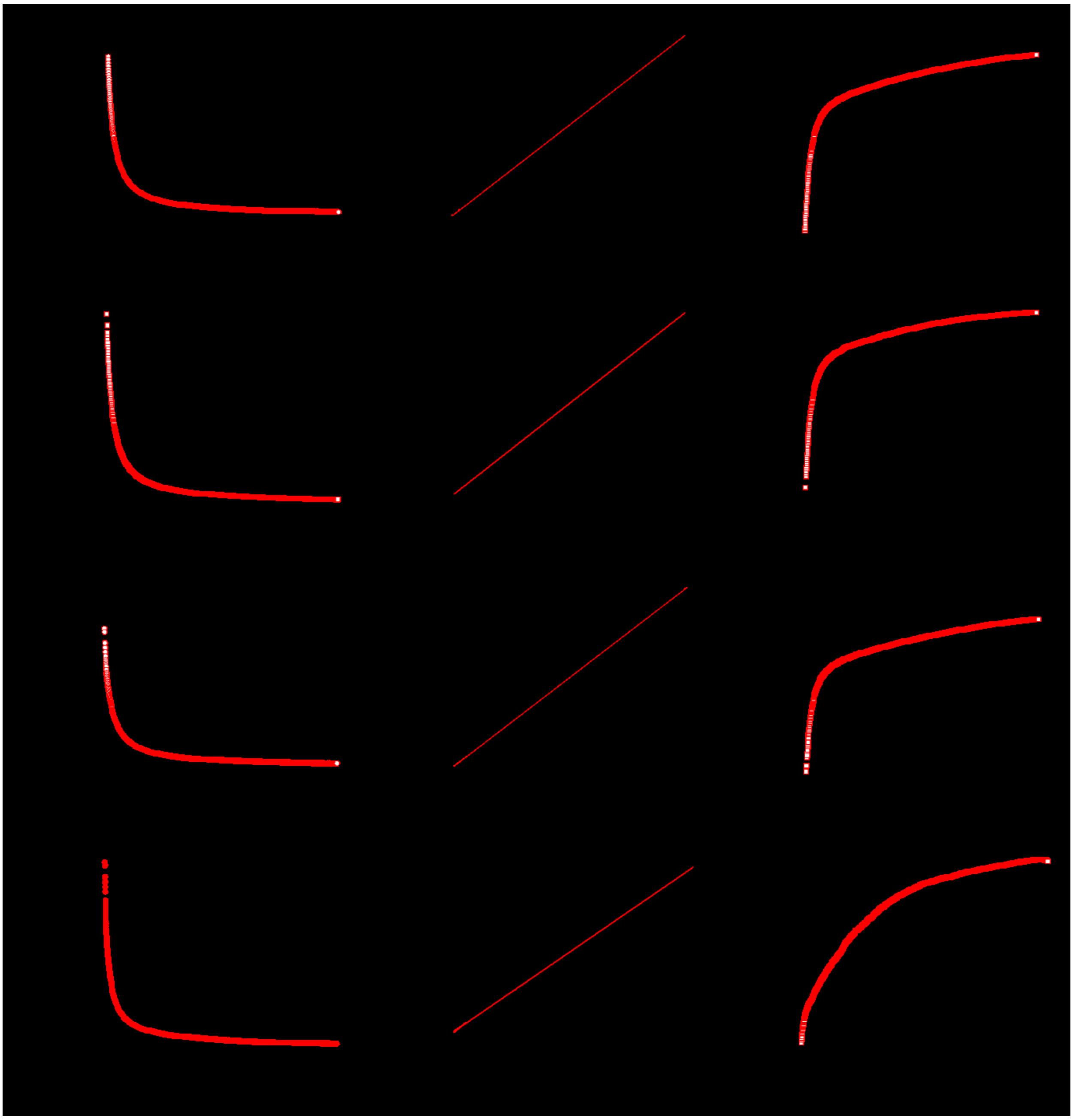

Figure S7 Temperature-dependent magnetization, Curie-Weiss fitting (red line) of the inverse mass susceptibility (*1/χ*) *vs. T*, and plot of $\chi_M T$ *vs. T* of (a–c) complex **1**, (d–f) complex **2**, (g, h, i) complex **3**, and (g–l) complex **4**, respectively.

The magnetic behaviors of complexes **1**, **2**, **3**, and **4** could be described by the Curie–Weiss law:

$$\chi = \frac{C}{T-\theta} \qquad \text{(eq. 1)}$$

where χ is mass susceptibility (emu g^{-1}); C is the Curie-Weiss constant (emu K g^{-1}); T is temperature (K); θ is the Weiss constant (K). The mass susceptibility of the compound is related to the molar susceptibility (emu mol^{-1}), χ_M:

$$\chi_M = \chi \, . MW/Z \qquad \text{(eq.2)}$$

where MW is the molecular weight of the compound and Z is the number of moles of magnetic ions per formula weight of the compound (for the calculation, the chemical formulae $C_{486}H_{456}N_{60}O_{274}Ni_{52}$ for **1**, $C_{540}H_{588}N_{60}O_{286}Ni_{52}$ for **2**, $C_{468}H_{394}N_{60}O_{270}Ni_{52}Br_{18}$ for **3**, and $C_{510}H_{490}N_{54}O_{278}Co_{52}$ for **4** are used).

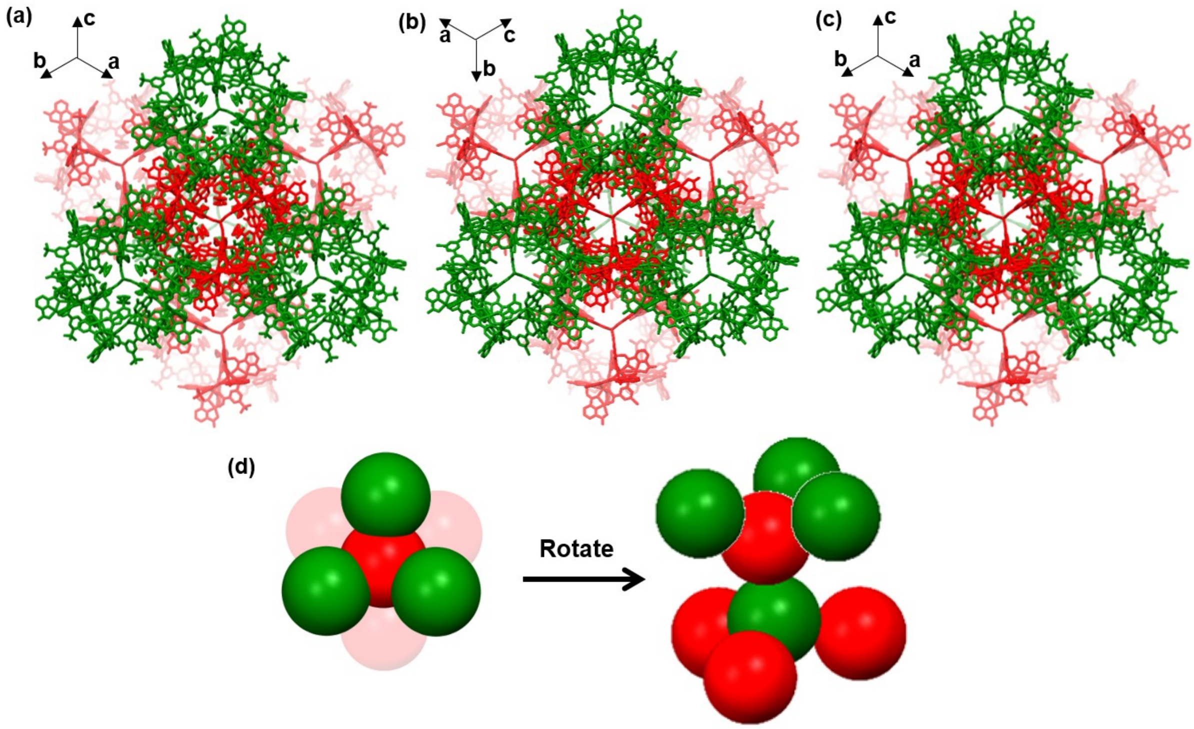

Figure S8 Packing of (a) **2**, (b) **3**, (c) **4**, (d) cartoon demonstrating the arrangement of M_6 and P_6 cages in space from different view.

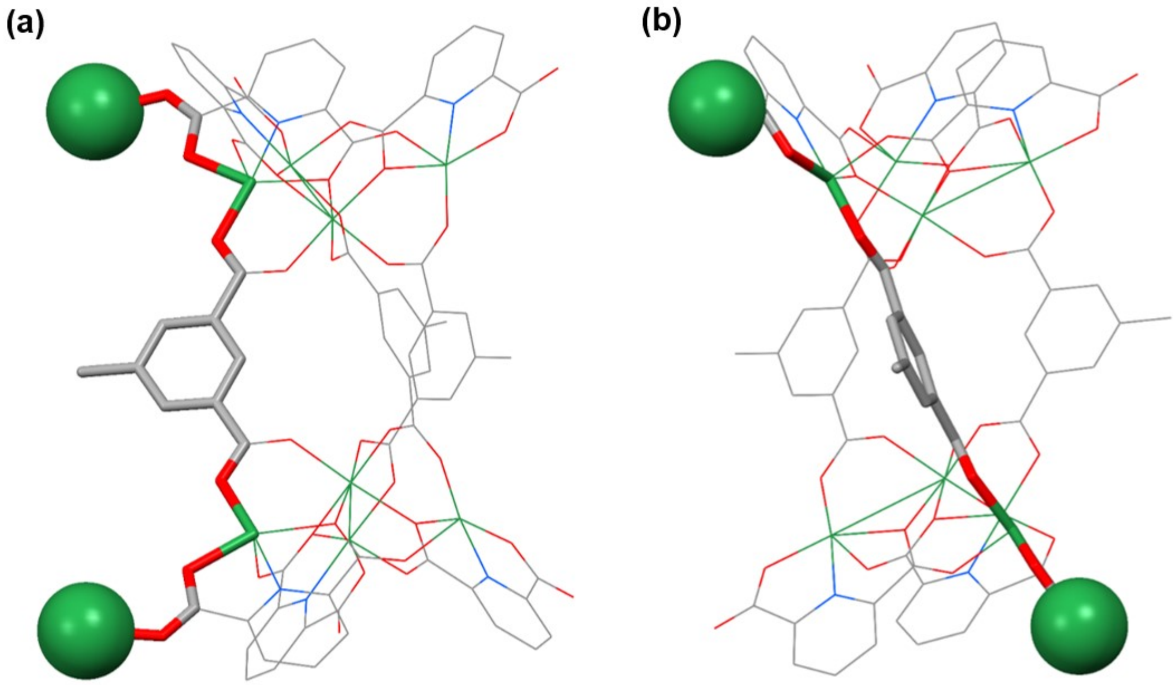

Figure S9 (a) The front view presentation of a building block with a “strand” created by a bridging ligand, connecting to two Ni05. (b) The view of the building block after being rotated 90° around its C_3 axis.

Figure S10
